# Laparoscopic median arcuate ligament release with an antegrade approach in adolescents and young adults: a single center experience

**DOI:** 10.1590/1806-9282.20250082

**Published:** 2025-09-19

**Authors:** Kutay Bahadir, Demet Sarıdemir Ünal, Selin Ural, Yunus Emre Sacın, Ayse Keven, Gungor Karaguzel, Ayhan Mesci

**Affiliations:** 1 Akdeniz University, School of Medicine, Department of Pediatric Surgery – Antalya, Turkey.; 2 Akdeniz University, School of Medicine, Department of General Surgery – Antalya, Turkey.; 3 Akdeniz University, School of Medicine, Department of Radiology – Antalya, Turkey.

**Keywords:** Abdominal pain, Celiac artery, Laparoscopy, Median arcuate ligament syndrome

## Abstract

**OBJECTIVE:**

Median arcuate ligament syndrome is a rare pathology where the median arcuate ligament causes compression on the celiac trunk. Different techniques are utilized in the treatment of median arcuate ligament syndrome, and studies on a standard laparoscopic technique are limited. The aim of this study is to evaluate the surgical and clinical outcomes of patients who underwent laparoscopic "antegrade" release of median arcuate ligament in a single tertiary center.

**METHODS:**

This retrospective study includes nine adolescents/young adults who underwent laparoscopy for median arcuate ligament syndrome between 2016 and 2024. All laparoscopic procedures were performed with an antegrade approach. The patients’ demographic data, symptoms, radiologic imaging methods, operative technique, and postoperative outcomes were recorded.

**RESULTS:**

There were seven female and two male patients in our series. The median age at diagnosis was 17 (range: 15–26) years. The most common symptoms were postprandial abdominal pain (n=9), nausea and vomiting (n=8), and weight loss (n=7). Doppler ultrasonography and/or computed tomography angiography (n=9, 100%) were performed in all patients as preoperative diagnostic imaging. The mean operation time was 92 (range: 60–110) min. The mean oral intake time was 1.2 (range: 1–3) days. The mean hospitalization time was 3 (range: 2–5) days. There was no conversion to laparotomy. One patient had a recurrence during follow-up and underwent a secondary intervention. The mean follow-up time was 62.6 (3–88) months.

**CONCLUSION:**

Laparoscopic median arcuate ligament release with an antegrade approach is safe and feasible to perform and adequate in terms of symptom relief and celiac artery compression release in both adolescent and young adult groups.

## INTRODUCTION

Median arcuate ligament syndrome (MALS)—also named as Dunbar syndrome or celiac axis compression syndrome—is a rare pathology where the median arcuate ligament (MAL) causes a compression on the celiac trunk^1,2^. The incidence of MALS is unknown; however, it seems to be increasing over the years as the use of radiologic imaging such as Doppler ultrasound (US), computed tomography angiography (CT angiography), and magnetic resonance imaging (MRI) becomes more widespread^2^.

Current treatment for symptomatic MALS cases is to decompress the celiac trunk by releasing the MAL either with an open, laparoscopic, or robotic-assisted surgical approach^3^. While open decompression is the traditional approach, laparoscopic and robotic-assisted approaches have also been used successfully, gaining more popularity over the years^3,4^. Laparoscopic and robotic release have the advantages of decreased postoperative pain, earlier onset of oral intake, and better cosmetic outcomes^5^. On the other hand, the robotic-assisted surgical approach is limited by higher costs^6^. Different techniques are utilized in the treatment of MALS, and studies on a standard laparoscopic technique are limited. Laparoscopic release of MAL can be performed by either an antegrade approach or a retrograde approach^7-9^.

The aim of this study is to share our experience on adolescents and young adults with MALS who underwent laparoscopic "antegrade" treatment in our center.

## METHODS

### Study design and data collection

The study was performed in adherence to the Declaration of Helsinki and was approved by the local ethics committee (No: 25.04.2024/TBAEK-270). A declaration of informed consent was received from all patients or parents of underaged patients. Nine patients who underwent laparoscopic release of MAL from 2016 to 2024 were retrospectively evaluated and enrolled in this study. All patients were operated in a single institution by the departments of pediatric surgery and general surgery. An open surgical approach and loss of records were criteria for exclusion.

Data on the patients’ demographic data, presenting symptoms, weight at preoperative evaluation, radiologic imaging results, operation details, postoperative feeding, length of hospital stay, recurrence, and follow-up results were collected retrospectively from the hospital database.

### Surgical technique

**Figure 1:**
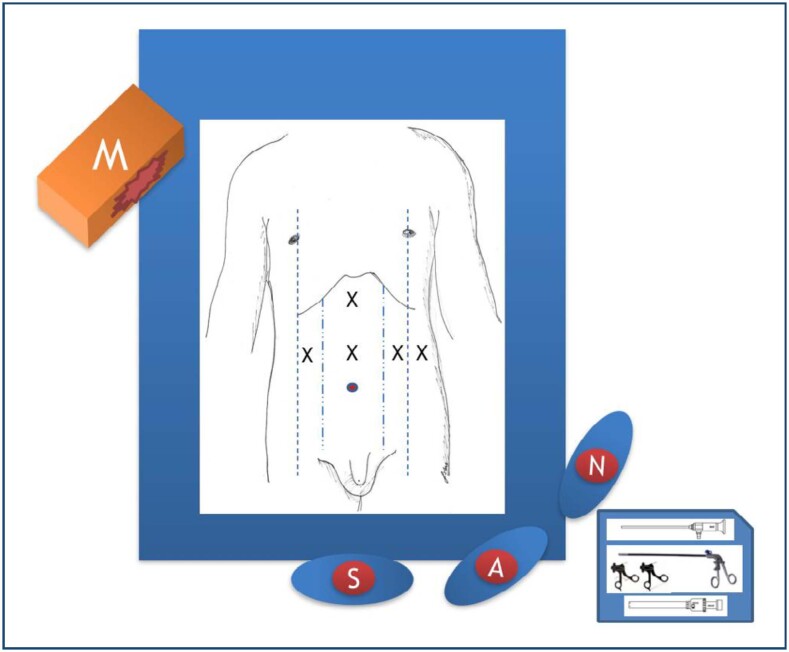
Surgical French position, the operating surgeon is placed between the patient's legs. Trocar placements can be seen marked with an "X."

**Figure 2:**
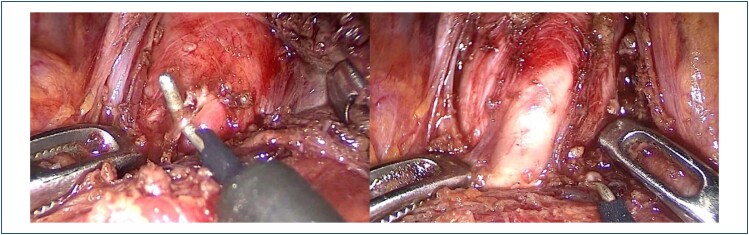
Images from the laparoscopic median arcuate ligament release procedure. (A) Median arcuate ligament ligation using hook cautery. (B) Skeletization of the celiac trunk at the end of the procedure.

The patients were placed in reverse Trendelenburg French position while the operating surgeon stood between the patient's legs (Figure 1). A 10 mm camera trocar was inserted into the umbilicus or 5 cm superior to the umbilicus (the decision was made in the operation room), and carbon dioxide was insufflated to achieve 12 mmHg pressure in the abdominal cavity. Laparoscopy was performed with a 30° video laparoscope, which was used through the camera trocar. The operation was performed through the insertion of three additional trocars under direct vision: a 10 mm trocar on the midclavicular line of the left upper abdomen, a 5 mm trocar infralateral to this port, and a 5 mm trocar on the midclavicular line of the right upper abdomen. A Nathanson retractor was inserted through another 5 mm epigastric incision for liver retraction. The gastrohepatic ligament was divided by a harmonic scalpel to identify the right crus of the diaphragm. The esophagus was dissected cranially to the level of the esophageal hiatus, and the esophagus was retracted along with the cardia and gastric fundus. This stomach retraction, both caudally and laterally, allowed access to the aorta. In our antegrade approach, dissection was started on the aorta, and the diaphragmatic crus was dissected starting cranially. Dissection followed caudally until the celiac trunk was localized by the poststenotic dilation and pulsation on the anterior surface of the aorta. The celiac trunk was carefully skeletonized to relieve the compression, and the surrounding fibrous tissue was also ligated to achieve adequate decompression (Figure 2).

### Statistical analysis

Descriptive statistics were used to describe the overall study population. The mean, median, and standard deviation were estimated.

## RESULTS

Nine cases were operated for MALS with a laparoscopic antegrade approach. There were seven (77.8%) females and two (22.2%) males aged from 15 to 26 years; the median age was 17±4.35 years. The most common presenting symptom was postprandial abdominal pain (n=9, 100%); additional signs and symptoms included nausea and vomiting (n=8, 88.8%), weight loss (n=7, 77.8%), and diarrhea (n=2, 22.2%) (Table 1). Comorbidities included chronic antral gastritis (n=6, 66.7%), gastroesophageal reflux (n=6, 66.7%), asthma (n=1, 11.1%), hypothyroidism (n=1, 11.1%), and mitral valve insufficiency with infective endocarditis (n=1, 11.1%).

**Table 1:** Patients’ data.

Patient no.	Age at the time of operation (year)	Sex	Presenting symptoms	Weight loss (kg)/time (months)	Duration from the onset of symptoms to MALS diagnosis	Preoperative imaging	Initiation of enteral feeding (day)	Length of hospital stay (day)
1	15	F	Postprandial abdominal pain, nausea and vomiting, diarrhea	5/1	1	US, CT	3	5
2	15	M	Postprandial abdominal pain	7/2	2	CT	1	2
3	16	M	Postprandial abdominal pain, nausea and vomiting	12/8	8	US, CT	1	4
4	16	F	Postprandial abdominal pain, nausea and vomiting	17/8	8	US, CT	1	2
5	17	F	Postprandial abdominal pain, nausea and vomiting, diarrhea	7 / 18	18	US, CT	1	2
6	17	F	Postprandial abdominal pain, nausea and vomiting	6/3	3	US, CT	1	3
7	22	F	Postprandial abdominal pain, nausea and vomiting	-	43	CT	1	4
8	25	F	Postprandial abdominal pain, nausea and vomiting	-	27	US, CT	1	2
9	26	F	Postprandial abdominal pain, nausea and vomiting	10/14	14	CT	1	3

MALS: median arcuate ligament syndrome; US: ultrasound; CT: computed tomography.

Diagnostic imaging in the preoperative period was performed for all patients: Doppler US (n=6, 90 66.7%) and CT angiography (n=9, 100%).

Laparoscopy was the initial approach for nine patients. All procedures were completed laparoscopically. The mean operation time was 92 min (range: 60–110 min). The mean feeding initiation time was 1.2 days (1–3 days). The mean hospitalization time was 3 days (2–5 days). The mean follow-up time was 62.6 months (3–88 months).

## DISCUSSION

MALS syndrome is a rare pathology caused by the compression of the celiac trunk by the fibrotic diaphragmatic crus^10^. This study presents the outcome of nine patients diagnosed with this rare pathology who underwent laparoscopic MAL release with an antegrade approach.

MALS generally presents in middle-aged females; however, cases of adolescents and children have been reported^9,10^. Metz et al presented a review study of six pediatric and 38 adult patients with a mean age range of 15–17 and 30–61 years, respectively^11^. We preferred to enroll adolescents and young adults in the present study to obtain more objective data from a homogeneous population. Six patients who underwent laparoscopic MAL release were in the adolescent age group (12–18 years), and three patients were in the young adult age group (19–26 years). Female predominance was present in the population of this series, as is consistent with the literature. The greatest challenge in the treatment of MALS patients is the difficulty of the diagnosis, which is usually elusive and one of exclusion^12,13^. Another challenge is that there is no uniform criterion for the diagnosis of MALS, which consists of clinical symptoms, radiologic imaging, and exclusion of other pathologies^13,14^. Thus, patients have long-standing symptoms with various indeterminate investigations for other etiologies such as gallbladder disease, peptic ulcer, appendicitis, inflammatory bowel disease, and mesenteric ischemia^12,14,15^. Differentiation of MALS from chronic mesenteric ischemic pathologies is especially more challenging since both have overlapping symptoms with delayed diagnosis, so it is commented in the literature that MALS might be underdiagnosed^15^. In this series, the mean time of diagnosis after onset of symptoms was 13.77 months (range: 1–43 months), which emphasizes the delay of diagnosis discussed in the literature.

**Figure 3:**
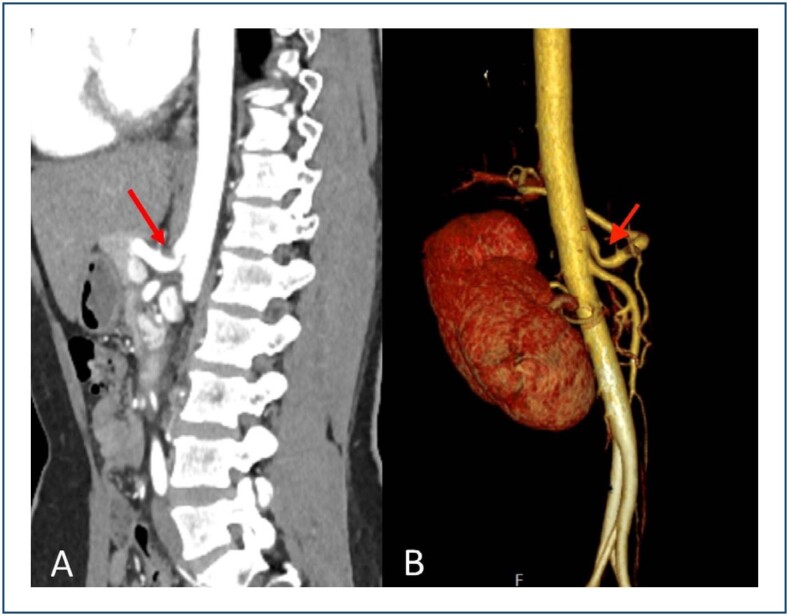
(A) Sagittal arterial phase computed tomography angiography image and (B) volume-rendered 3D reconstruction from abdominal aortogram. The hooked appearance of the proximal celiac axis (red arrow) with poststenotic dilatation is being caused by the median arcuate ligament indenting upon the superior aspect of the artery.

Doppler US is the initial diagnostic test for MALS as it is inexpensive, without radiation, readily available, and can provide the peak inspiratory and expiratory velocity along with the image of the stenotic vessel^9^. In the literature, peak systolic expiratory velocity greater than 350 cm/s and bending of the celiac trunk greater than 50˚ are considered as signs of MALS^14^. CT angiography is another imaging technique used for MALS diagnosis^14^. It has been a reliable indicator for MALS since the development of processing technologies that provide a clear 3D image of the vascular structure, allowing for a diagnosis of MALS along with all the aortic branches (Figure 3)^14^. However, CT angiography is generally performed during the inspiratory phase, while MALS is more prominent during the expiratory phase^14^. Selective catheter angiography is another technique utilized for MALS, with the disadvantage of being invasive, making it a less preferred choice for diagnosis^16^. In our practice, the general approach to patients with symptoms suspicious of MALS was initially by Doppler US and/or CT angiography. In our series, one patient with prominent symptoms who had a normal finding on CT angiography underwent a celiac angiography, which revealed a 70–90% obstruction in the celiac trunk.

Once the diagnosis of MALS is established, determining the most appropriate treatment remains complex and debated. While endovascular techniques such as stenting or angioplasty have demonstrated success in the management of mesenteric ischemia, their efficacy in cases of extrinsic vascular compression—such as in MALS—appears limited^16^. These interventions are associated with higher rates of long-term failure and recurrence, which has relegated them to a secondary role in the treatment of MALS, particularly in adult patients^16^. Moreover, there is currently no evidence evaluating the safety or effectiveness of these approaches in the pediatric population.

Release of MAL in treatment can be performed with an open or minimally invasive approach^16^. Laparoscopic decompression of the celiac artery is used as a minimally invasive technique for the treatment of MALS^17^. On the other hand, laparoscopic surgery recognized advantages such as avoiding midline incisions, shorter hospital stays, reduced postoperative pain, and earlier resumption of oral intake^5^. Laparoscopic antegrade or retrograde approaches can both be utilized with the aim of sufficient exposure and the adequate release of MAL without any vascular injury^7-9^. In the antegrade approach, fibrous bands are dissected starting from the diaphragmatic crus, and the dissection is followed caudally, while in the retrograde approach, dissection is started from the left gastric artery and continued cephalad until the celiac trunk is completely exposed and release of fiber bands is achieved^7,9^. Unfortunately, no studies have been published about the standardized and commonly accepted method for the laparoscopic techniques for MALS treatment. In this series of nine cases, all operations were completed with an antegrade laparoscopic approach, and there was no conversion to laparotomy due to inadequate exposure and vascular injury. However, a large series of MALS patients who underwent laparotomy as reported by Reilly et al. demonstrated that MAL release when performed with celiac revascularization had a lower rate of recurrence (24 vs. 44%)^18^. Khrucharoen et al. and Jimenez et al. reported symptom recurrence in laparoscopic MAL release as 30.8 and 38%, respectively^18,19^. In our series, symptom recurrence following surgery was seen in three patients. In the literature, the recurrence rate of laparoscopic MAL release is approximately 6–18%^3,4,18^. In our laparoscopic series, only one case of recurrence occurred.

Reports indicate that laparoscopy provides greater immediate postoperative symptom relief compared to open surgery^20^. However, according to the literature, one-third of these patients have recurrence during follow-up^19^. Whether the recurrence is due to an inadequate surgical release or progression of the currently unknown cause of MALS, both the diagnosis and management of recurrence in these patients present a challenge^19^. Furthermore, pediatric MALS is highly associated with additional psychiatric disorders, reported to be as high as 50% in the literature. The association of these disorders with MALS symptoms and their severity is unclear, but the need for psychological evaluation and intervention for pediatric MALS patients is debated^21^. In our series, three patients described the continuation of symptoms. After re-evaluation with CT angiography, one was diagnosed with MALS recurrence and was managed with a secondary intervention; two had normal CT angiography findings and were referred to the department of psychiatry. These two patients had relief of symptoms with psychiatric referral and follow-up. This may be explained by the impact of psychiatric disorders on MALS.

This study had the limitations of being a retrospective observational study. The study did not present any questionnaire or survey data. Also, due to the single-center nature of the study, the sample size was small, as is expected of a rare pathology such as MALS. Another limitation is that, due to the lack of a comparative group of MALS patients operated with an open approach or laparoscopic retrograde approach under the same settings as our series, it is not possible to draw a reliable conclusion comparing open and laparoscopic approaches.

In conclusion, MALS is a rare diagnosis with challenging management. Lack of systematic diagnostic criteria and treatment algorithms in a very small group of patients presents additional difficulties when faced with a decision for surgical treatment. Although we have a limited number of patients, our tertiary center experience has supported that the antegrade laparoscopic approach in both adolescents and young adults is a safe and effective technique with a low recurrence rate in the treatment of MALS.

## ETHICAL APPROVAL

Written informed consent was given by the patient's parents/legal guardians. This study was approved on 25.04.2024 by the institutional ethical committee (TBAEK-270by). The study was carried out in accordance with the Declaration of Helsinki.

## Data Availability

The datasets generated and/or analyzed during the current study are available from the corresponding author upon reasonable request.
